# Event-Driven Deep Learning for Edge Intelligence (EDL-EI) †

**DOI:** 10.3390/s21186023

**Published:** 2021-09-08

**Authors:** Sayed Khushal Shah, Zeenat Tariq, Jeehwan Lee, Yugyung Lee

**Affiliations:** 1 Department of Computer Science and Engineering, University of North Texas, Denton, TX 76207, USA; zeenat.tariq@unt.edu; 2 College of Architecture, Myongji University, Seoul 03674, Korea; jeehwanlee@mju.ac.kr; 3 Department of Computer Science and Electrical Engineering, University of Missouri, Kansas City, MO 64110, USA; leeyu@umkc.edu

**Keywords:** edge intelligence, event-driven deep learning, IoT intelligent system, sensor fusion, air-quality event

## Abstract

Edge intelligence (EI) has received a lot of interest because it can reduce latency, increase efficiency, and preserve privacy. More significantly, as the Internet of Things (IoT) has proliferated, billions of portable and embedded devices have been interconnected, producing zillions of gigabytes on edge networks. Thus, there is an immediate need to push AI (artificial intelligence) breakthroughs within edge networks to achieve the full promise of edge data analytics. EI solutions have supported digital technology workloads and applications from the infrastructure level to edge networks; however, there are still many challenges with the heterogeneity of computational capabilities and the spread of information sources. We propose a novel event-driven deep-learning framework, called EDL-EI (event-driven deep learning for edge intelligence), via the design of a novel event model by defining events using correlation analysis with multiple sensors in real-world settings and incorporating multi-sensor fusion techniques, a transformation method for sensor streams into images, and lightweight 2-dimensional convolutional neural network (CNN) models. To demonstrate the feasibility of the EDL-EI framework, we presented an IoT-based prototype system that we developed with multiple sensors and edge devices. To verify the proposed framework, we have a case study of air-quality scenarios based on the benchmark data provided by the USA Environmental Protection Agency for the most polluted cities in South Korea and China. We have obtained outstanding predictive accuracy (97.65% and 97.19%) from two deep-learning models on the cities’ air-quality patterns. Furthermore, the air-quality changes from 2019 to 2020 have been analyzed to check the effects of the COVID-19 pandemic lockdown.

## 1. Introduction

For a healthy life, sophisticated techniques are needed to observe and identify air-quality levels. The Internet of Things (IoT) and artificial intelligence play a crucial part in enhancing the quality of care and sustaining urban growth. Social identity is one of the critical determinants for user satisfaction and ecological convenience when taking into consideration various environmental criteria such as greenhouse gases, air temperature, relative humidity, wind speed, and noise levels [1].

In addition, IoT and mobile computing have shown significant advances in the deployment of sensors and IoT devices and real-time data collection from diverse settings, such as environmental sensing and the monitoring of human activities via video/audio surveillance [2,3]. We are facing a lot of edge big data, with zillions of bytes of real-time data available at the network edge [4]. Artificial intelligence with deep learning has achieved remarkable success in various applications, including computer vision, Natural Language Processing, and real-time video/audio processing. However, advances in AI for IoT or large-scale sensor systems have been relatively slow. This is because of challenges in processing a massive amount of heterogeneous data in real time. Thus, the marriage of IoT and AI, edge intelligence (EI), is urgent and will receive tremendous interest if successfully developed. However, Zhou et al. have pointed out that research on EI is still in its infancy, and a collaborative effort from both computer systems and AI for the advances of EI is required [4].

There are significant challenges in the processing of many sensors [5] that provide millions of readings a second. How to understand meaningful circumstances with real-time constantly changing data in the spatial–temporal domain is an open question. Imagine that the data come not from a single sensor but from multiple diverse sensors. Each produces a varying range of data, and distribution patterns of the value for these sensors are quite different from sensor to sensor. Various applications built with IoT and sensors include healthcare for discovering anomalies and symptoms for disorders, diseases, and epidemics [6], traffic monitoring and control [7], and agriculture and livestock farming [8]. However, there has been very little work on the integration of AI and IoT, such as work by Chen et al. [9] on controlling and coordinating traffic lights in large-scale urban networks.

Edge-computing-based deep learning is highly restricted compared to cloud-based deep learning. This is because the edge has no computing capability to build super-powerful deep-learning models. Additionally, although powerful models can be made in the cloud, they are too big to be deployed in the edge, or sometimes they are too general to apply to specific issues that may arise in the edge. Thus, small and context-aware models are suitable for the edge. For example, recent work has presented an edge-computing-based deep-learning model based on the fog/edge-computing paradigm [10]. Additionally, some recent deep-learning solutions have shown interesting applications, including a hand-gesture authentication system with edge-based deep learning [11].

Demand for edge intelligence (EI) has been vital in various domains such as the smart city, the smart grid, and healthcare. However, it is not easy to deal with real-time IoT sensor data from multiple streams. These data streams are complex dimensions of space, time, and frequency domains and need to be fused and processed together to generate successful machine-learning tasks. In addition, as big neural networks may not be suitable for edge devices, it is necessary to consider the building of a small contextual model that supports processing data streams from IoT devices and many sensors. In particular, some studies present novel advances in edge intelligence, such as environmental sensing for traffic control in a smart city [12], human activity recognition using wearable devices [13], air-quality detection and forecasting [14], and workload prediction in a smart grid [15]. However, there is still room to improve for scalable and effective deep learning with edge devices. For example, predictive models could be built based on the contexts of observed events in the domains of interest.

Edge Intelligence may show great potential application for real-time monitoring or detection of environmental issues. Lin et al. [16] presented smart medical robots and multi-sensor fusion technologies for building human–robot interaction models for real-time monitoring in health care. Cai et al. [17] presented the detection of environmental conditions and dynamic obstacles for an autonomous driving system in multiple domains such as urban and rural areas, traffic densities, weather, and times of the day. However, these solutions are specific to a particular context and are not generally applicable to other contexts.

State-of-the-art research mainly focuses on time-series forecasts in specific contexts. Some advanced edge-intelligence (EI) techniques have thus been proposed to deal with real-world problems. However, to our knowledge, there is little work investigating how real-world problems are solved based on an event-driven deep-learning approach in edge intelligence. We proposed an innovative solution, which will contribute to improving the understanding and applications of deep learning in EI research. The major difference of our approach compared to the proposed work is discussed in the Related Work section (see Table 1). The contribution of this study is summarized as follows:

- We propose a novel event-driven deep-learning edge-intelligence (EDL-EI) framework that aims to support cloud and edge collaboration.
- An event-driven detection model was designed to demonstrate large sensor fusion for event detection using edge-based deep-learning models.
- We designed an IoT prototype system with multiple sensors and edge devices for real-time event detection.
- We have developed the proposed an EDL-EI framework by designing complex events regarding air quality and traffic congestion. To demonstrate the event model, we have used multiple sensor datasets captured from real settings of multiple cities and different timelines, including during the COVID-19 pandemic.
- We have conducted extensive experiments with the datasets. Our experimental results confirm the feasibility and effectiveness of our approach, which achieves outstanding classification accuracy and time-series forecasting in air-quality prediction in the edge considering specific temporal and geospatial contexts.

## 2. Related Work

### 2.1. Edge Intelligence

Edge intelligence [4] has received a lot of attention. However, the research has still not been fully explored. Deng et al. [23] sought to present potential study prospects. The first addressed the relationship between edge computing and AI (artificial intelligence). AI for edge or AI on edge is expressed in a centralized architecture. The research separates edge computation scientific research into topology, content, and service using the bottom-up method, and presents some instances of empowering the edge with understanding. It divides the investigations in AI on edge into modeling adaption, framework design, and processing amplification using a top-down breakdown, and provides some current research achievements. Furthermore, it describes the cutting-edge and significant problems in numerous hot themes for AI for edge and AI on edge.

In DeepSense [13], IoT data fusion techniques were proposed to leverage spatiotemporal relationships by integrating CNNs for multiple sensor modalities and RNNs (recurrent neural networks) for the understanding of temporal correlations in various IoT applications such as car-tracking, human activity recognition, and biometric identification.

Zhou et al. [4] introduced Edgent, a participatory and on-demand DNN (deep neural network) co-inference architecture with device–edge interaction. Agents identified new architectural parameters to tailor the latencies of a DNN model towards low-latency edge cognitive ability: DNN segmentation, which enables collaboration among edge and mobile devices; and DNN right-sizing, which molds the overall processing need for a DNN. Initially, deployment and assessments on Raspberry Pi show that Edgent effectively allows low-latency edge intellectual ability.

Guillen et al. [24] developed an edge-computing remedy. They assessed edge computing for frost forecasting in agricultural products using Long Short-Term Memory (LSTM) deep neural networks to estimate colder temperatures. LSTM deep neural networks are particularly challenging, but they produce exceptional outcomes when forecasting statistical analysis. Their best-proposed model achieves a difference of fewer than 1 ${}^{\circ }$C after being trained with three months of data, 2500 iterations, and 150 receptors. Its processing time is less than five hours, allowing training before actual prognostication.

Our work is different from the previous works as follows: DeepSense [13] is a cloud-based solution, but it is not suitable for edge computing. However, our EDL-EI system enables models to be deployed for real-time event detection of events on the edge. Zhou et al. [4] presented an interesting co-inference architecture with device–edge interaction. They used agent technology to facilitate the interaction while we used optimized event-detection models mostly handled by the edge without help from the cloud. Compared to Guillen et al. [24]’s work, our findings show that innovative edge-computing technologies, such as NVIDIA Jetson Xavier’s low-power graphics processing unit (GPU), could be helpful in edge computing, primarily as a credible option for intelligent systems.

### 2.2. Large-Scale Sensor Fusion for Real-Time Event Detection

Lin et al. [16] presented human–robot interaction models for real-time monitoring using intelligent medical robots and multi-sensor fusion technologies. The multi-sensor fusion method was designed based on an interpretable neural network for body sensor networks based on communication and data processing with various sensors and robots in medical human–robot interactions. Cai et al. [17] presented an autonomous driving system using end-to-end control for autonomous vehicles in various environmental conditions and dynamic obstacles. For this study, the modeling of cars, pedestrians, motorcyclists, and bicyclists had been conducted based on diverse data from multiple domains such as urban and rural areas, traffic densities, weather, and times of the day from cameras, LiDAR, and radar. Balemans et al. [25] proposed knowledge-based pruning based on a sensor fusion method for autonomous driving applications. Mainly, they focused on a neural network optimization method for resource efficiency with a sensor fusion approach for edge devices. Interestingly, layer-wise relevance propagation (LRP) was proposed for the localization of network knowledge using the pruning method.

Wang et al. [22] presented an event-detection approach using deep-learning models for power system situation awareness and event investigation. They designed two convolutional neural networks and fused them to improve detection accuracy by over 48% with short detection time. Kidzinski et al. [26] presented automatic real-time gait-event detection using sing Long Short-Term Memory (LSTM) in a pathological setting. They reported that the event-detection model is suitable and sufficient for detecting variability in kinematics and anatomy of patients, and due to the real-time predictions and controls, it has great potential for assistive devices for patients. Choi et al. [27] proposed real-time event detection for apnea–hypopnea using convolutional neural networks and a single-channel nasal pressure signal. They have obtained 96.6%, that is higher than the average accuracy (94.9%) of sleep apnea and hypopnea syndrome (SAHS) diagnosis and reduced event-detection time.

There is a significant difference in our work from these previous works on real-time event detection: Lin et al. [16] demonstrated their robots and multi-sensor fusion technologies in a medical setting. The significant difference between their work and ours is that we proposed image-based deep learning as scalable multi-sensor fusion while using feature-based deep learning. Cai et al. [17] mainly focused on probabilistic model building (without considering edge intelligence) with visual data from cameras, LiDAR and radar, and environmental conditions. In contrast, we focus on edge-based event-detection modeling using visual analytics with real-time sensor data. Balemans et al. [25] proposed a knowledge-based approach for sensor fusion while using an event-based approach with multi-sensor fusion technologies to establish edge intelligence. Wang et al. [22]’s event-driven deep -learning approach is similar to our work. However, we focus more on the detection of events using optimized models for edge intelligence. Kidzinski et al. [26] and Choi et al. [27] presented significant use cases of real-time event detection in healthcare settings. However, they did not show the great potential of edge intelligence.

### 2.3. IoT/Sensor Innovation and Machine Learning for Air-Quality Prediction

Marin et al. [28] offered a cost-effective solution to costly municipal facilities. They examined the placement of detectors in a variety of locations throughout cities to measure the quality of the air. Researchers discovered that enough data were gathered from sensors in diverse fields and that the information was stored on a remote server. Nevertheless, the results have become less dependable, leading to a shortage of sufficient sensor calibration. They concluded that converting current remote monitoring from regional facilities to a sensor/cloud is a difficult task. Nonetheless, in terms of versatility, the sensor/cloud environment is a cost-effective option.

Kumar et al. [29] suggested a hardware-based method for the calculation of environmental parameters. To visualize sensor values and transmit information to the IBM Bluemix Remote server, the system contains a Raspberry Pi coupled to Node-Red. Researchers evaluated it on equipment in Delhi, India, and found that low-cost sensors provided a more aesthetically pleasing output than Doppler radar readings. Zhen et al. [30] created a sensor network-based hardware solution for air-pollution measurement-platform sensors linked together via a low-cost sensor network to interact with a cloud-based server. The real-time air-quality monitoring system was evaluated against the government air-quality station in the area, and an interface for end users with air-quality filtration was created.

Regarding the IoT and sensor design, Marin et al. [28] built a cost-effective solution for air-quality measurements across different municipal facilities. However, they faced issues with sensor calibration at different locations resulting from the centralized data-gathering process. However, our edge-based system is cost-effective and learns on-site in feasible environments to work more efficiently than a cloud-based approach.

Kumar et al. [29] performed data visualization for air quality using Raspberry Pi and Node-Red, which is low-cost equipment, and compared the results to Doppler radar readings. Similarly, Zhen et al. [30] created low-cost hardware in the cloud for the comparison of low-cost sensors across governmental stations. Our approach demonstrated that low-cost IoT/edge platforms could be integrated with deep learning to establish edge intelligence in the performance of advanced predictions.

Han et al. [31] used machine learning to predict the air-quality index (AQI) for cities in the United States. Investigators used Weka [32,33] to examine data and generate a machine-learning algorithm to predict 2 significant harmful emissions: nitrogen dioxide (NO2) and ground-level ozone (O${}_{3}$). In terms of MAE (mean absolute error) and RMSE (root mean square error) values, research found that the support vector machine outperformed other machine-learning approaches. Chen et al. [34] used physical sensors to investigate the air pollution of 16 large metropolitan areas. They identified the substances that impacted the air quality then established a link between them. Furthermore, they applied PEK-based machine learning (an ensemble artificial neural network (ENN)-based hybrid function approximator), and projected the air0quality level with 22 shared essential elements. A PMI (partial mutual information)-based IVS (input variable selection) approach for air quality has been used to evaluate the factors. The findings revealed that PM25, PM10, and SO${}_{2}$ are the most key denominators impacting the air-quality index and are still closely associated.

The detection of air pollution in urban areas was carried out by Xi et al. [20]. The WRF-Chem model allowed them to analyze the situation and develop a better forecasting model that can be modified in a timely manner. The researchers created a complete architecture using features from the WRF-Chem model. The proposed structure assisted in a higher level of forecast improvement than the WRF-Chem forecasting model. They took into account 74 Chinese cities. They applied support vector machines, decision trees, random forests, and gradient boosting, all of which are machine-learning models. Their precision ranged from 70 to 85% for the ten leading cities, ranging from 40 to 53% for the bottom ten cities.

Raj et al. [21] employed low-cost sensors for a systematic air-quality assessment undertaken across Denmark’s smart cities. Artificial neural networks, support vector machines, and multiclass support vector machines were used to do binary and multiclass classification using machine-learning methods. They claimed that SVM outperformed ANN. Moreover, the polynomial SVM accomplished the highest of all, detecting anomalies at a rate of around 95%.

The works by Han et al. [31], Chen et al. [34] Xi et al. [20], and Raj et al. [21] for the prediction of air quality are limited since they rely on traditional machine learning with individual sensor readings. Unlike that work, we proposed a low-cost solution for the detection of different chemicals at city level using time-series data transformation into images. We made the data suitable for a proper deep-learning method to classify the binary and multiclass classifications. Our predictive accuracy is much higher than what they reported.

## 3. Proposed Approach: Deep-Learning Models for Event Detection

### 3.1. Architecture of Edge Intelligence

**Figure 1:**
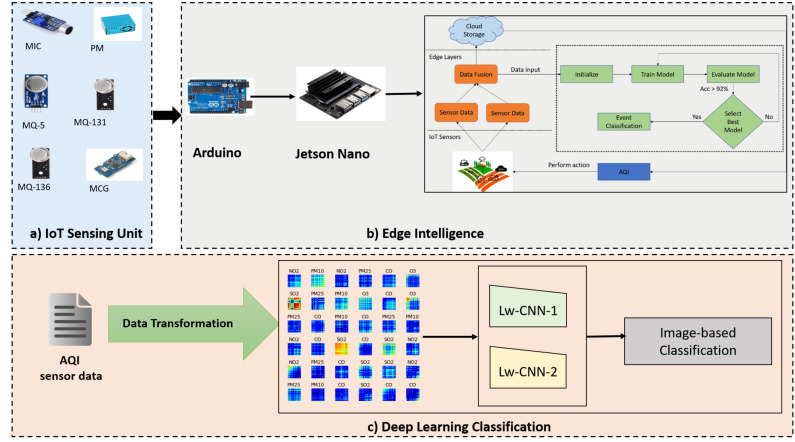
Architecture of event-based deep-learning framework for edge intelligence (EDL-EI).

We now discuss the system architecture of the proposed event-driven deep learning for edge intelligence (EDL-EI). The EDL-EI framework consists of sensors, micro-controllers, and the deep-learning server components. As seen in Figure 1, there are three main things: the sensing unit, edge intelligence, and deep-learning classification. Each of them is described below.

*Sensing Unit*: Sensors for tracking air-quality levels (AQI) are included in the sensing unit. We selected the MQ gas sensors [35] to identify the toxic compounds in the atmosphere because of their size, price, and dependability as an initial concept. Table 2 shows that the MQ-5 sensor has been used to measure carbon monoxide (CO), the MQ-131 sensor for ground-level ozone gas detection, the MQ-136 sensor for SO${}_{2}$, the MQ-135 gas sensor for nitrogen dioxide, and the PMS5003 sensor for particulate matters (PM10 and PM2.5) (PM10 and PM2.5). The sensors are linked to Arduino, a fully accessible digital prototype technology that allows people to create dynamic electronic equipment. Analog connections are used to interface the sensors to the Arduino board. Regarding duration and frequency, Table 2 presents the limits and measurements for an air-quality data recorder. The analog data collected by the sensors is sent via the Arduino to the NVIDIA Jetson nano for digital signal computation.

*Arduino Uno*: The Arduino Uno is a microprocessor that is developed on the Microchip ATmega328P microprocessor [36]. The Arduino Uno is a low-power, open-source board with analog and digital pinouts related to specific I/O connectors. It runs on 9 V batteries. Arduino provides technologists with an integrated development environment (IDE) for developing applications with sensor input via digital and analog connections. To expand their capabilities, the Arduino Uno can be used in conjunction with many other integrated devices, such as the Raspberry Pi and the NVIDIA Jetson Nano. Within our approach, data from the Arduino board’s sensors is collected over a physical interface and evaluated in a much more sophisticated edge device, the NVIDIA Jetson Nano, enabling real-time machine learning. In our proposed system, Arduino’s role is to provide an analog gateway for MQ sensors to send their data directly to Jetson Nano for digital processing.

*Jetson Nano*: NVIDIA Jetson Nano [37] is indeed a small, capable, intelligent computer that includes the main machine-learning technologies such as TensorFlow, Keras, and PyTorch, and allows for high-performance AI application execution. The NVIDIA Jetson Nano is compact, low-power, and low-cost, using a microprocessor chip based on the ARM architecture. A high-quality GPU video card is included on the motherboard, allowing for high-performance computation. The Nano, as with the Raspberry Pi, has GPIO (general-purpose input/output) ports. The Jetson Nano can support several Linux-based programs, but it really is best known for its edge-computing capabilities, which include real-time machine learning. In comparison to Raspberry Pi or even CPU-based existing systems, GPU-based architecture would easily manage machine-learning operations. Our system’s major part is the Jetson Nano, which records analog data from Arduino in a database server. The information acquired would be used for machine learning. The NVIDIA Jetson Nano houses the MySQL server and machine-learning server, which are all used to do real-time machine-learning projects.

*Deep-Learning Classification*: The deep-learning server runs typically on Jetson Nano in our system. The purpose of the system is to take the time-series data from the sensors and automatically apply data transformation to convert the data into images. Furthermore, it has two convolutional neural networks (CNNs) to classify the data into concerned classes every 24 h. According to standard AQI calculation, we take 24-h windows to have accurate results compared to the official standard. The CNN architecture of the proposed deep-learning models is shown in Figure 1.

### 3.2. Deep Learning for Event Detection

We proposed an event-driven deep learning for edge-intelligence (EDL-EI) framework that can handle complex events with real-time sensor datasets, the preprocessing of real-world data considering events of interest, the design of lightweight deep-learning models, and the deployment of an IoT prototype system. Some important features of the EDL-EI framework are summarized as follows:

- It defines an event (starting time, ending time, conditions, stages, stage changes, actors, environmental factors, etc.) for indicating a domain of interest. It identifies relevant parameters that could be contributed for defining an event. Additionally, it determines dominant parameters using correlation analysis.
- It conducts data-preprocessing-relevant data from sensors or edge devices and converts them into an appropriate format (sensor data into images). It provides guidelines on building deep-learning models and connecting such models that are lightweight and suitable for real-time event detection.
- It deploys the models to the edge devices, for which we developed the IoT-based prototype system with multiple sensors and NVIDIA Jetson Nano. It uses the models for real-time event detection and notification on the edge.

#### 3.2.1. Event-Detection Model

An event-based approach was designed to specify an event of interest. In this paper, we apply correlation analysis to identify the dominant parameters for a specific event. For example, there are lots of parameters to define an air-quality event, including natural gas, LPG, ground-level ozone, CO, ammonia, benzene, alcohol, smoke, hydrogen sulfide gas (SO${}_{2}$), particulate matters (PM10 and PM2.5), and digital temperature/humidity sensor to measure the surrounding air. For another example of traffic events, we may consider several parameters, such as time, traffic load, latitude, longitude, height from sea level, humidity, and temperature. How do we define a complex event, which is common in real-world real-time contexts, considering multiple events? To the complex event, we need to find the relationships between two different events, such as air-quality events and traffic events.

Another unique property of our event model is its ability to detect events in real time using deep-learning models available on the edge. The lightweight deep-learning models are built with context-aware datasets considering the dominant parameters we identified from the correlation analysis. Since the models should be small enough for the edge devices, selecting the parameters for the training data is critical. Thus, we claim that lightweight deep learning is built according to the event method.

**Figure 2:**
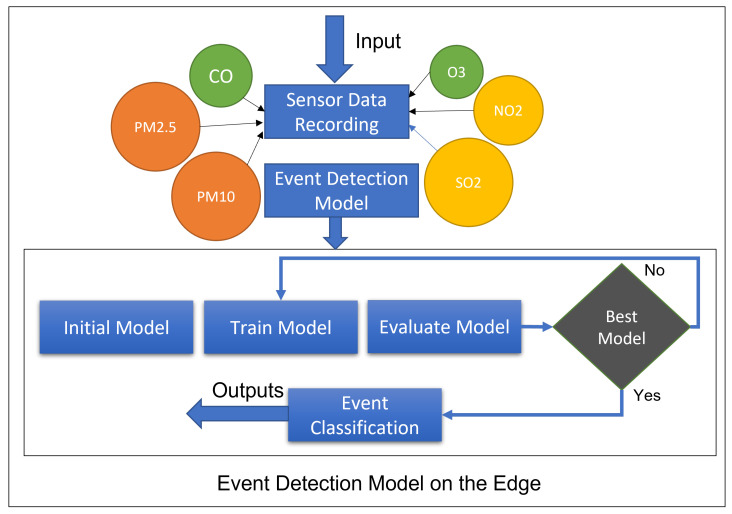
Event-detection model.

As an example, we consider a traffic event that has a significant effect on the environment. Figure 2 shows the overall working of the event detection in our system using deep learning that is performed on the edge. Different sensors are deployed at different locations to detect the concentration for each gas individually, and the sensor data fusion is applied to convert the data into images that will be used in deep learning. Once the deep-learning model is built, it will be deployed to the edge to ensure real-time event detection can happen in near real time considering user contexts.

**Figure 3:**
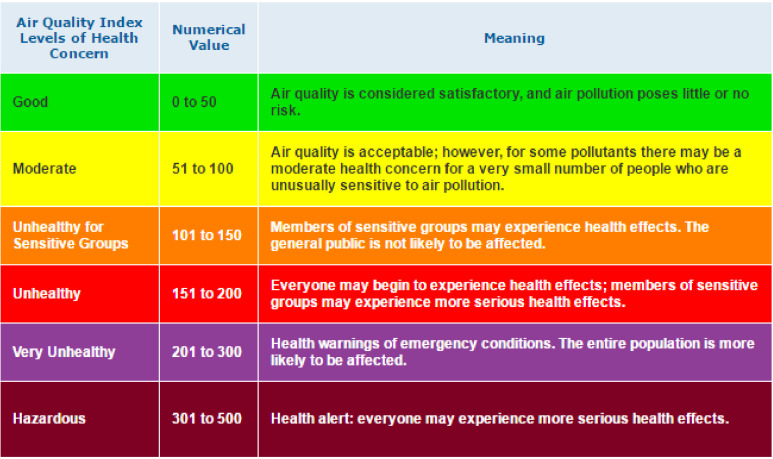
Official air-quality index measurement.

We also define complex events considering multiple events, such as air-quality events and traffic events. The generic flow of the event-detection model for air-quality event (AQI) is shown in Algorithm 1. The event-detection algorithm initializes an event with the conditions and duration of the event and detects when any condition is changed. For example, traffic events and air-quality events are monitored during the 24-h timeframe, and we check if any specific condition has been changed, such as traffic increase, or PM2.5 increase. This event will be detected as the conditions are defined for the event of the interest. Furthermore, from this particular situation, we know that these two events are strongly correlated, or one event (increased traffic flow) could be a cause of another event (decreased air quality). More specifically, the selection service module component is using the AQI estimation formula for calculation of the side-index importance of every pollutant after obtaining the ambient air-intensity predicted values, and to pick the maximum significance of the side-index valuation as the legitimate AQI value and correlate this to the air-quality index table shown in Figure 3. Algorithm 1 depicts the decision-making stages of this module.
**Algorithm 1** AQI for Event Detection**Input :** D ← Data recorded from different sensors over Time in seconds(Ts)
Input: M ← Set of instances with a set of highly detected AQI values mass level L
**Parameters:** ← Record data every minute,
Select highest value every hour,
∀ append one highest value every hour for 24 h
**Generate:** Images using Gramian Angular Field (GAF).
Output: E ← Array to store values for effect of event on AQI and area, event start time
Ts and end time Te
Initialization E ←clear(E), i ← 0
 **foreach**
*a* in *A* do
  **for**
*l* ← *L* do
   **if**
*D* [*a*] > *M*[*l*] then
     *E*[*i*] ← *D*[*a*] and *A*[*a*]
      *i* ← *i* + 1
      **break**
    **endif**
  **endfor**
 **endforeach**
**Forall** events (sensor data recordings) that have entered the list for detection Adjust Ts and Te Merge ∀ events ← Ts or Te overlap Ts (Start time) and Te (End Time) ← Update event list **end for**


#### 3.2.2. Data Transformation

Deep learning’s breakthrough in computer vision and voice recognition has fueled the deep-learning boom. Building prediction models a time-series, on the other hand, may be arduous. Recurrent neural networks are harder to train, research is less relevant, and there are no pre-trained models available [38]; 1D-CNN may be cumbersome. To capitalize on the methods and insights of recent advances in computer vision, we will present and explore one way to encode time-series as images based on the Gramian Angular Field (GAF) [38].

The inner product is a function that compares the “similarity” of two vectors. It enables the application of conventional Euclidian geometry concepts such as length, angles, and orthogonality in dimensions 2 and 3. The product of two vectors *u* and *v* in the simplest instance (2D space) is defined as:

(1) $$\langle u,v\rangle =u_{1}\cdot v_{1}+u_{2}\cdot v_{2}$$

and it may be demonstrated that:

(2) 〈u,v〉=∥u∥·∥v∥·cos(θ)

As a result, if *u* and *v* seem to be of normalization 1, we obtain:

(3) 〈u,v〉=cos(θ)

As a result, when working with unit vectors, their inner product is defined only by the angle (given in radians) between *u* and *v*. Furthermore, the resultant value falls inside the range [−1, 1]. In Euclidean settings, the formal method of the product of *u* and *v* is shown as follows:

(4) $$\langle u,v\rangle =\sum_{i=1}^{n}u_{i}\cdot v_{i}$$

The Gram matrix is a helpful tool in linear algebra and geometry. It is often used to calculate the linear dependency of a collection of vectors, among other things. The Gram matrix of a group of n vectors is a matrix formed by the dot-product of each pair of vectors (see the similarity). Mathematically it can be represented as:

(5) $$G=\left(\begin{array}{cccc} \langle v_{1},v_{1}\rangle & \langle v_{1},v_{2}\rangle & \ldots & \langle v_{1},v_{n}\rangle \\ \langle v_{2},v_{1}\rangle & \langle v_{2},v_{2}\rangle & \ldots & \langle v_{2},v_{n}\rangle \\ \vdots & \vdots & \ddots & \vdots \\ \langle v_{n},v_{1}\rangle & \langle v_{n},v_{2}\rangle & \ldots & \langle v_{n},v_{n}\rangle \end{array}\right)$$

Similarly, supposing that all 2D vectors have always had the same norm, we obtain:

(6) $$G=\left(\begin{array}{cccc} \cos\left(\phi_{1,1}\right) & \cos\left(\phi_{1,2}\right) & \ldots & \cos\left(\phi_{1,n}\right)\\ \cos\left(\phi_{2,1}\right) & \cos\left(\phi_{2,2}\right) & \ldots & \cos\left(\phi_{2,n}\right)\\ \vdots & \vdots & \ddots & \vdots \\ \cos\left(\phi_{n,1}\right) & \cos\left(\phi_{n,2}\right) & \ldots & \cos\left(\phi_{n,n}\right) \end{array}\right)$$

The GM maintains spatial dependence. Because time rises from top-left to the lower end, the time dimension is stored in the matrix’s shape. Any solution set will eliminate material about either of the two images of perception. Therefore, we create a new input x operation, $x⊕y=\cos\left(\phi_{1}+\phi_{2}\right)$

(7) $$G=\left[\begin{array}{cccc} \cos\left(\phi_{1}+\phi_{1}\right) & \cos\left(\phi_{1}+\phi_{2}\right) & \cdots & \cos\left(\phi_{1}+\phi_{n}\right)\\ \cos\left(\phi_{2}+\phi_{1}\right) & \cos\left(\phi_{2}+\phi_{2}\right) & \cdots & \cos\left(\phi_{2}+\phi_{n}\right)\\ \vdots & \vdots & \ddots & \vdots \\ \cos\left(\phi_{n}+\phi_{1}\right) & \cos\left(\phi_{n}+\phi_{2}\right) & \cdots & \cos\left(\phi_{n}+\phi_{n}\right) \end{array}\right]$$

**Figure 4:**
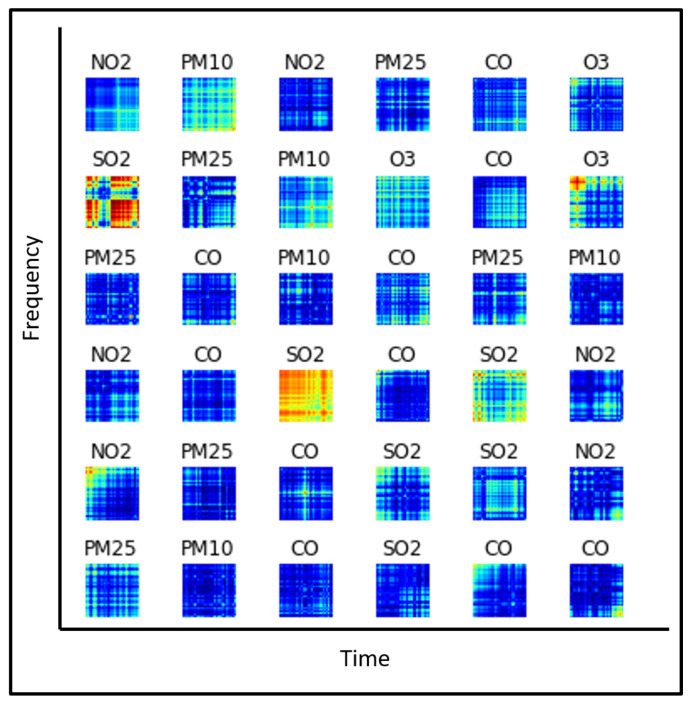
Image feature visualization for multiple sensor time series.

Thus, we transform the time-series data into images using the above operation. Some examples for images generated in different patterns for different sensors are shown in Figure 4.

#### 3.2.3. Feature Extraction for Multi-Sensor Fusion

To classify different sensor patterns based on individual city, we have considered data with records from two years, i.e., 2019 and 2020, for multiple sensors of PM2.5, PM10, CO, NO${}_{2}$, SO${}_{2}$, and O${}_{3}$. Initially, we obtained data in column-separated format for two years with 365 records per year which are further available for each hour on every date. We combined the data in 24 h format to restrict our experimentation to the AQI official standard [39]. Secondly, we applied data transformation as explained in the previous section. After getting the image data, we converted all images to grayscale format using the CV2 library and resized the images to 128 × 128. Finally, we saved the features using CV2 into a NumPy array saved in float format to pass it as an input to the convolutional neural network for training, testing, and validation. Data converted into float data type is in a form to which we can apply one hot label encoder to test the “y_test” set and “X_train” set. Finally, we evaluated our model using the same labeled features.

### 3.3. Deep-Learning Modeling

The convolutional neural network (CNN) has been acknowledged as a popular and influential deep neural network model in image classification applications. We have developed two novel 2D-CNN models that will be feasible to implement on the transformed time-series data obtained in images. Our models are efficient and lightweight. Hence, they are easily deployed to edge platforms, i.e., Jetson Nano. Our model is a 2D-CNN model composed of an input layer, convolutional 2D layer, max-pooling layer, and fully connected layers. Every layer is followed by batch normalization and ReLU activation function and 25% dropout to avoid any overfitting issues.

The mathematical form of the convolutional layers is given in Equations (8) and (9)

(8) $$\left[x_{i,j,k}^{l}=\sum_{a}\sum_{b}\sum_{c}w_{i,j,k}^{\left(l-1,f\right)}y_{i+a,j+b,k+c}^{\left(l-1\right)}+{bias}^{f}\right]$$

(9) $$\left[y_{i,j,k}^{l}=\sigma \left(x_{i,j,k}^{\left(l\right)}\right)\right]$$

$y_{i,j,k}^{l}$ denotes the final output, where i,j,k is the three-dimensional input vector. The parameters for the filters are given by $w_{i,j,k}^{\left(l\right)}$, while the sigmoid activation equation for sequential activation is described by $\sigma (x_{i,j,k}^{\left(l\right)})$. The final step illustrated by Equations (10) and (11), is entirely interconnected.

(10) $$\left[x_{i}^{\left(l\right)}\sum_{j}w_{i,j}^{l-1}y_{j}^{l-1}+{bias}_{j}^{l-1}\right]$$

(11) $$\left[y_{i,j,k}^{l}=\sigma \left(x_{i,j,k}^{l}\right)\right]$$

**Figure 5:**
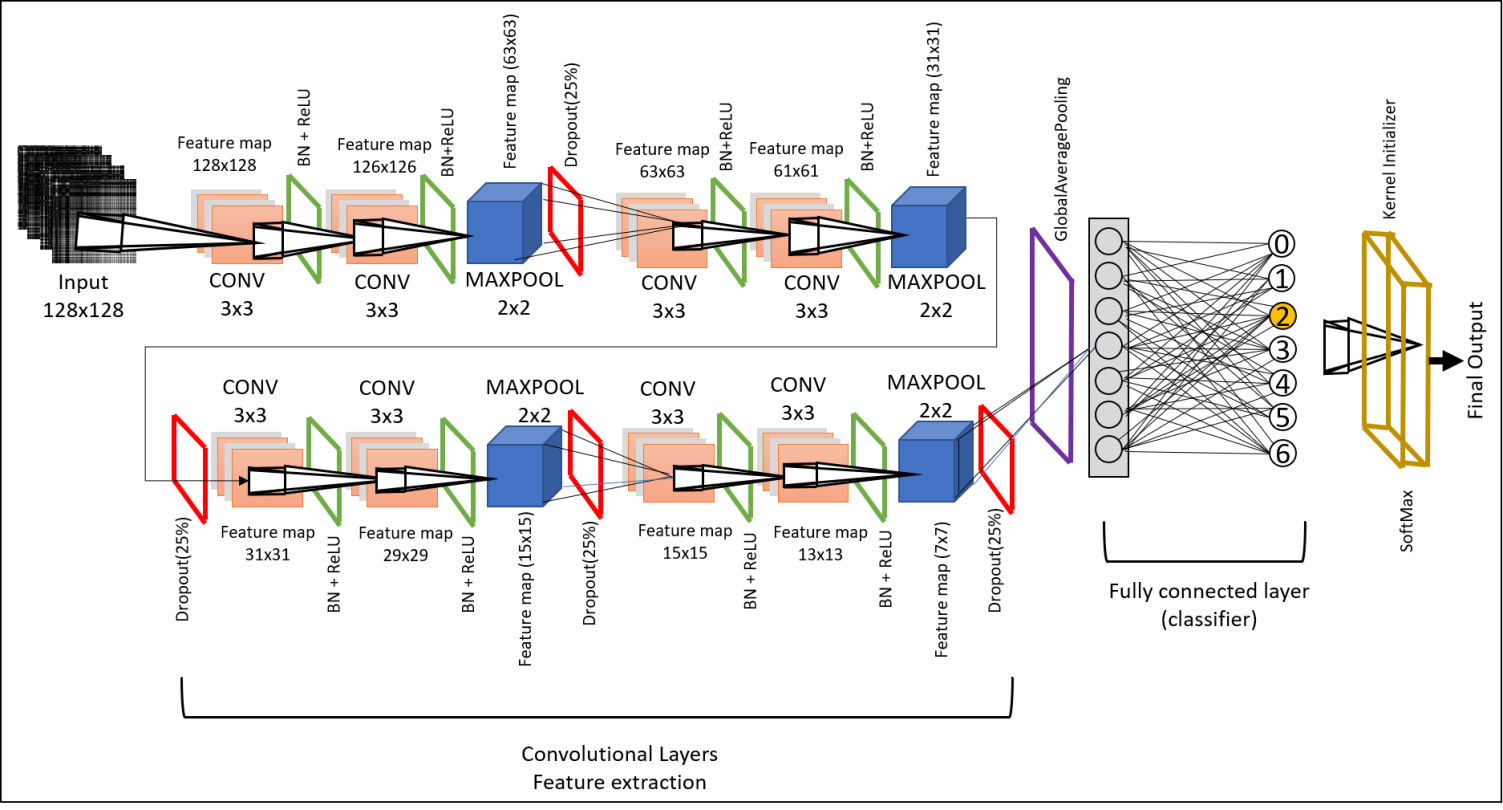
Lightweight CNN-1: convolutional neural network architecture for city-based air-quality prediction.

We named our model lightweight CNN-X (Lw-CNN-1 and Lw-CNN-2). The Lw-CNN is motivated by its lightweight parameters to make it as small as possible so it can be deployed easily to all edge devices and keep model efficiency in consideration. The model parameters and training time is given in Table 3. The image is provided in shape $X\in R^{128\times 128}$. The Lw-CNN-1 model comprises eight convolutional layers followed by a final dense layer that consists of the output. Each convolutional layer is followed by batch normalization. Batch normalization provides the facility to work independently. Additionally, it provides support to normalize the output of the previous layer to the next layer. A max-pooling layer follows every two convolutional neural layers. We have added a dropout of 25% to avoid model overfitting. The layer details, receptive field, feature maps, and output are shown visually in Figure 5. The total number of trainable parameters is 363,882 (0.36 M), and the time taken for training a model in NVIDIA JETSON Nano with 15 epochs is around 9 min and 38 s.

**Figure 6:**
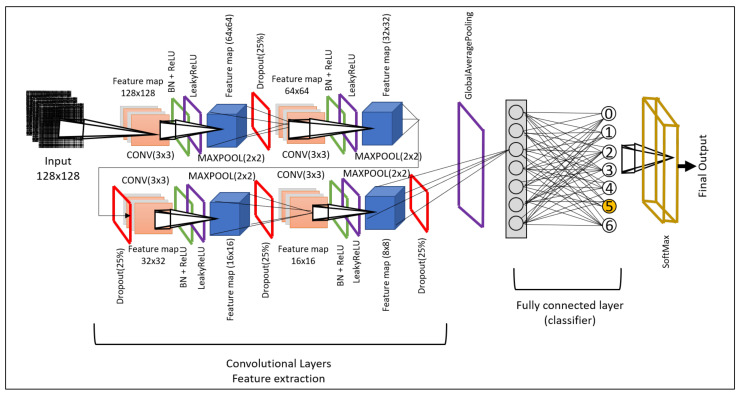
Lightweight CNN-2: convolutional neural network architecture for city-based air-quality prediction.

Classification of the transformed time-series data into images was scaled to X $\in R^{128\times 128}$ size and provided as input to the model. Lw-CNN-2 is composed of 4 convolutional layers followed by batch normalization, LeakyReLU, max pooling, and dropout. We have used ReLU on the convolutional layer and LeakyReLU again to ensure we do not lose any information. LeakyReLU is used to obtain negative information, but it is scaled to a minimal value, i.e., multiplied by 0.01. Next, we use batch normalization to normalize the previous layer for the upcoming layer. Then, max pooling is used to discard unneeded features and pass on only the required features, eventually decreasing the number of trainable parameters. Finally, we used “GlobalAveragePooling” to scale down the number of trainable parameters further. The visual architecture is shown in Figure 6. This model has a total of 132,042 (0.13 M) trainable features, which makes this model very light, even compared to our first model, Lw-CNN-1. The approximate time taken for training using 15 epochs to classification output is 8 min and 41 s.

During the model-building phase, we realized the importance of hyperparameters, i.e., BatchNormalization, reducing the model complexity and improving learning speed. As a result, ReLU and LeakyReLU produce faster and superior results. In addition, dropout is used to overcome the model under-fitting or overfitting during the learning process. Each of these is explained below:

- Batch Normalization: Batch normalization is a methodology for standardizing system inputs deployed to either the activations of a previous layer or the feeds themselves. Batch normalization reduces model complexity by speeding up learning (in some situations by decreasing or bettering the iterations) and providing some attention mechanism.
- ReLU: The rectified linear activation function, or ReLU for brief, is a spline vector quantity that outputs the input directly if the source is affirmative; otherwise, it outputs zero. Although a network that uses it is faster to train and frequently produces superior results, this has established the preferred activation function for several artificial neural networks.
- Dropout: The dropout layer mainly helps minimize regularization and changes input units into 0 as arbitrary with a certain regularity at every training phase throughout the training phase. It is worth noting that the dropout layer applies if the learning parameter is found suitable, which means that no data are lost during interpretation when working with a model.
- LeakyReLU: Leaky ReLU, in contrast to ReLU, is much more highly “symmetrical”, and so might overgrow. In such a case, the outcome is sometimes not predictable. Leaky ReLU is not necessarily better than regular ReLU, and it must always be used as a last option.

### 3.4. IoT System Design for Edge Computing

We have developed a prototype system as an edge platform for the EDL-EI framework with an NVIDIA Jetson Nano and a set of low-cost gas-emission sensors coupled to an Arduino microcontroller. This system was designed as a cloud and edge collaboration using a client–server architecture. The edge-computing platform was built with IoT devices such as sensors, Arduino microcontroller, and NVIDIA Jetson Nano. In addition, a cloud computing platform was used for the server for deep-learning modeling.

**Figure 7:**
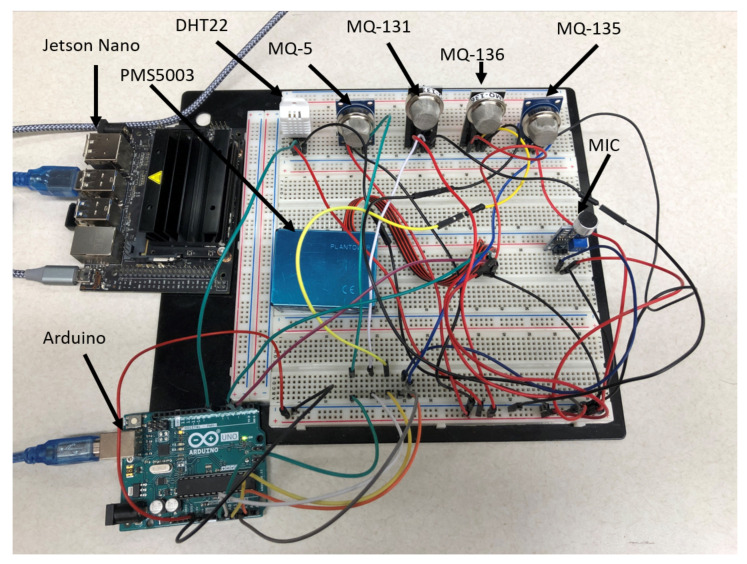
IoT Prototype circuit design.

A WLAN adapter connects the IoT module to the cloud server, allowing for more versatile and real-time detection in the proposed air quality and traffic event applications. The deep-learning modeling will occur throughout the cloud, while real-time event detection will happen in the NVIDIA Jetson Nano on edge. The data obtained from sensors on the edge will be retained internally for later use if the network is inaccessible. Figure 7 depicts our proposed prototype system.

We demonstrated that the suggested IoT sensing design for the EDL-EI’s edge platform obtains the superior accuracy of real-time event detection while providing a relatively low expense for the simple incorporation of numerous sensors. The edge platform design’s real-time event detection could enhance gas and pollution levels while maintaining the detectors’ reliability at desirable tiers throughout diverse environmental situations. Furthermore, the entire proportions have also been reduced to 13 × 11 inches in width and height. Thus, there are further benefits to its convenience and ease of perceived usefulness. The hardware, including sensors, Arduino, and the NVIDIA Jetson Nano, costs around $350. The solution was built using open-source IoT and real-time machine-learning initiatives.

## 4. Case Study: Influence of the COVID-19 Event on Air Quality/Traffic

### 4.1. Event Scenarios

In this paper, the COVID-19 pandemic event was set as a primary event. The air quality and associated effects, such as traffic due to the event, was analyzed. Event-driven deep-learning models were built to accurately predict temporal and spatial patterns regarding air quality and traffic congestion. Considering the pre-pandemic and the pandemic periods of 2019 and 2020 as shown in Table 4, we have analyzed how the different traffic patterns influence these relationships with the air-quality index (AQI), and how different parts of the world would be affected due to the pandemic event. Some studies show that the AQI level improved globally due to low gas emission during the COVID-19 lockdown [40].

To examine the effect of the COVID-19 pandemic on external environments such as air-pollution levels and traffic, counterfactual gas concentration in the air was considered the primary parameter. For this study, we considered both real-time data collected using our IoT system as well as the EPA’s dataset [41] during the pre-pandemic and pandemic periods in 2019 and 2020. In particular, we selected eight big cities with the highest pollution rates and critical pandemic situations: Wuhan, Beijing, Shanghai, and Hefei in China, and Seoul, Daegu, Incheon, Busan in South Korea. It was observed that there was a drastic change in the levels of AQI in China, and there was a small effect on South Korea’s AQI level. A significant difference in AQI was observed between 2015–2019 and 2020. Therefore, we compared 2019 and 2020 AQI in China for the two main cities, i.e., Beijing and Shanghai. The change in the level of AQI is affected mainly due to PM2.5. Road traffic is said to be the main contributor to the increase and decrease of PM2.5. We further performed our analysis on COVID-19. In 2020, a strict lockdown was imposed in China to control the outbreak. That was why we decided to conduct our study between 2019 and 2020 for the main polluted cities, i.e., Beijing and Shanghai. Upon our findings, we analyzed that the two most polluted cities were Beijing and Shanghai, among the other cities on which we were performing our analysis.

The relationship between AQI and traffic was further analyzed to show the event-based model with complex event scenarios. For the complex event detection, we used the data from the NASA Earth platform [42] including latitude, longitude, and different meteorological information about the environment concentrations, i.e., humidity, temperature, height, etc. We applied data fusion on EPA dataset [41] and the NASA MERRA-2 data [43] based on date, time, latitude, and longitude. MERRA-2 (Modern-Era Retrospective Analysis for Research and Applications- version 2) provides a viable ongoing climate analysis beyond MERRA’s terminus. A correlation between AQI data and different meteorological factors from the MERRA-2 dataset was further analyzed. The air-quality (AQ) forecasts are disseminated to the public through the AirNow.gov website [39], including the raw concentration index. We have cleaned the dataset with invalid values and applied data fusion to merge both datasets based on dates, latitude, and longitude.

### 4.2. Air-Quality Measurements

**Figure 8:**
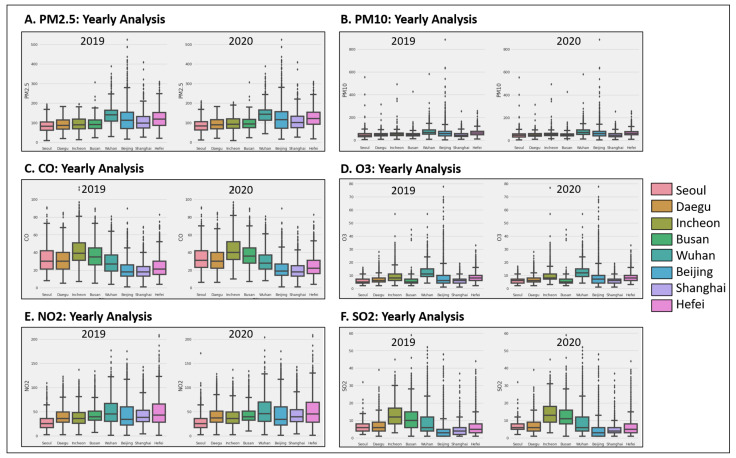
AQI level comparison for eight cities in China and South Korea before and during lockdown.

We have performed different air-quality measurements keeping COVID-19 as our primary source of discussion. COVID-19 is considered a global pandemic, and worldwide, millions of people are affected due to it. The first case was reported in December 2019. However, we kept in strict lockdown in the initial months of 2020. While the world was regaining control, the lockdown was eased in the middle and end of 2020. Before the pandemic outbreak began, everything was operating as usual, i.e., schools, offices, factories, and regular routines. It is to be noted that due to the implementation of lockdown, people were quarantined. Hence, there was a drastic decrease in traffic. Due to that decrease, the air quality improved. We can see from Figure 8 that 2019 has a high concentration for all gases in the environment as compared to 2020. We have performed our analysis on PM2.5, PM10, CO, O3, NO2, and SO2. There is an improvement from 2019 to 2020, considering all the sensors. In-depth analysis was performed for all cities mentioned above. There was a significant effect caused in the towns of China, i.e., Wuhan, Beijing, and Shanghai. However, a significant impact was noted in Beijing and Shanghai, specifically for PM2.5, O${}_{3}$, and CO.

**Figure 9:**
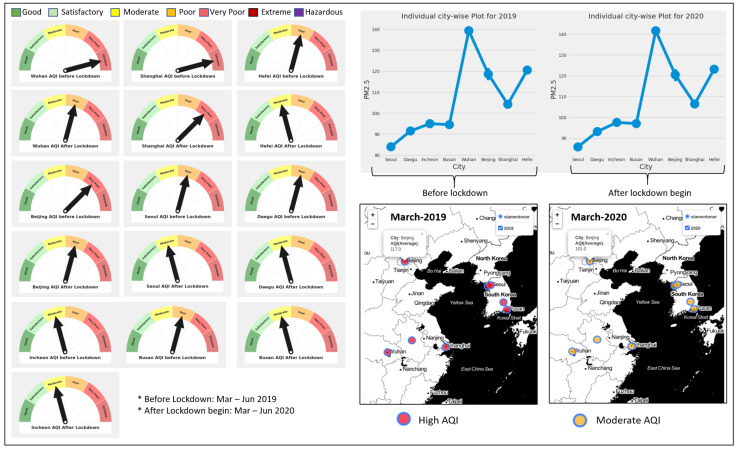
AQI level comparison for eight Cities in China and South Korea before and during lockdown.

The US Environmental Protection Agency (EPA) has set a standard for evaluating the AQI that can be seen in Figure 3. The formula for calculating the AQI according to the EPA standard is shown in Equation (12). According to this standard, we evaluated the data from the sources mentioned above for all eight cities. We observed the AQI before and after lockdown. The observation can be seen visually in Figure 9. The exact figure also demonstrates the city-wise PM2.5 concentration for 2019 and 2020 and the map visualization for the high AQI before lockdown. It has a very positive effect during the lockdown that was implemented.

(12) $$I=\frac{I_{\mathrm{high}\;}-I_{\mathrm{low}\;}}{C_{\mathrm{high}\;}-C_{\mathrm{low}\;}}*\left(C-C_{\mathrm{low}\;}\right)+I_{\mathrm{low}\;}$$

I: the (air-quality) indexC: the pollutant concentration$C_{\mathrm{low}\;}:$ the concentration breakpoint that is ≤C$C_{\mathrm{high}\;}$ : the concentration breakpoint that is ≥C$I_{\mathrm{low}\;}$ : the index breakpoint corresponding to $C_{\mathrm{low}\;}$$I_{\mathrm{high}\;}$ : the index breakpoint corresponding to $C_{\mathrm{high}\;}$$C_{\mathrm{low}\;},C_{\mathrm{high}\;},I_{\mathrm{low}\;}I_{\mathrm{high}\;}$ are from the US EPA Pollutant Breakpoint

### 4.3. Traffic Event Measurements

In this section, we considered traffic as an event and performed an analysis of two cities of China, i.e., Beijing and Shanghai. We considered these two main cities because, during our search, we found that Beijing and Shanghai were the two main polluted cities among all eight cities that we considered for COVID-19 analysis.

Our study was extended to incorporate the effects of the COVID-19 pandemic on air quality and traffic congestion. Traffic is considered to be the primary source of increases in PM2.5-related mortality in the environment. A relationship between air quality and traffic congestion can be determined by comparing the air-quality levels during the pre-pandemic and pandemic periods of 2019 and 2020. Due to lockdown, decreasing road traffic has resulted in an improvement to air quality.

**Figure 10:**
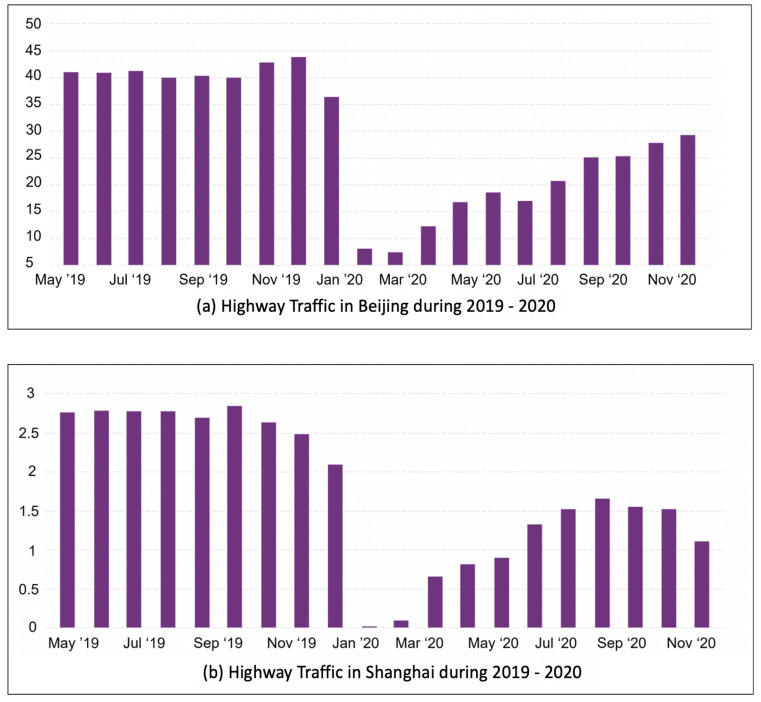
Beijing and Shanghai highway traffic: (**a**) 2019 (**b**) 2020.

**Figure 11:**
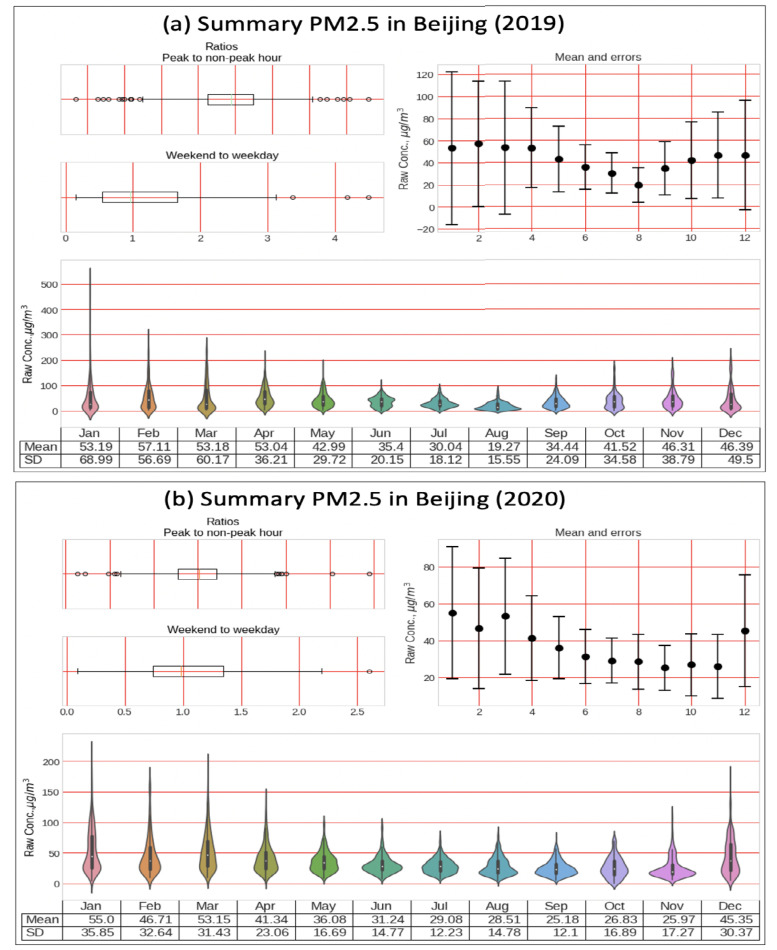

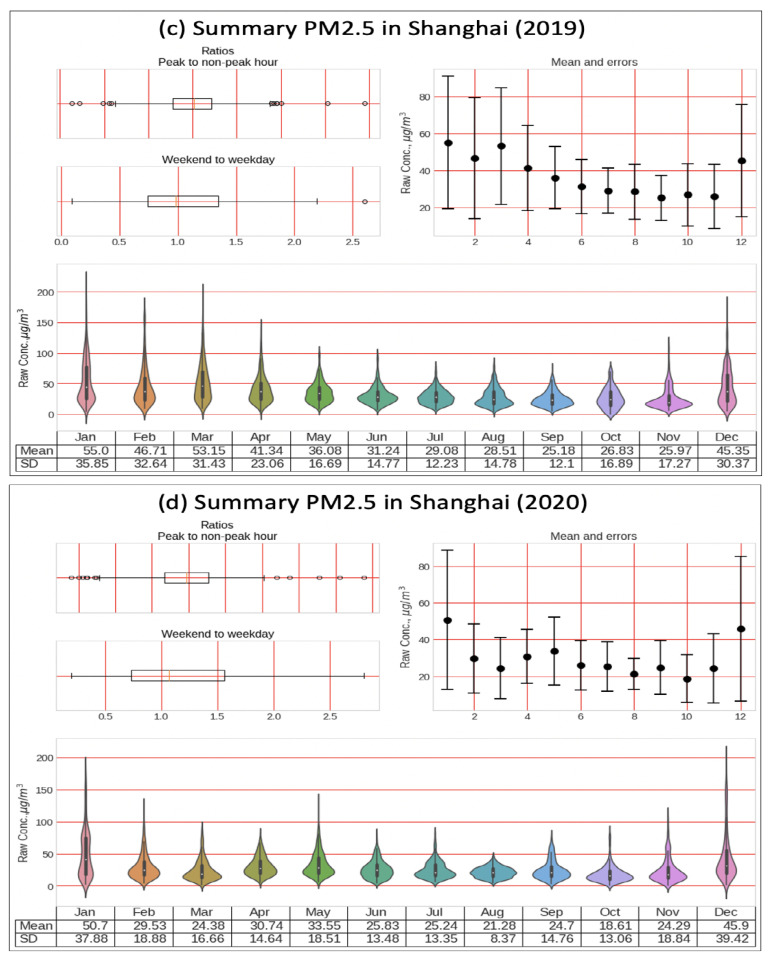
Air quality & traffic event, PM2.5 ratio for peak/non-peak hours: (**top**) Beijing in 2019 and 2020 (**bottom**) Shanghai in 2019 and 2020.

According to the Department of Transportation China (CIEC), the highest passenger volume traffic on highways in 2019 was 41.2, whereas in 2020, the highest person volume was recorded as 29.38, which was recorded in December, while the lowest person volume per million was recorded as 16.81 in May 2020. Looking at the highest average, we can say that air quality has improved due to the imposed lockdown in Beijing due to COVID-19. Similarly, in Shanghai, the highest recorded volume of traffic was 3 in 2019 and 1.6 in 2020, as shown in Figure 10. In our analysis, we have used data from the US Embassy [39]. They have reported air-quality data for the six main types of sensors for different years. Our analysis reports that the AQI has a direct relationship with traffic. As the traffic for the cities above increased, the AQI also increased, and at points where a decrease was noticed, the AQI improved and vice versa. Furthermore, working on a data-cleaning process, we have also reported the raw concentration for peak vs. non-peak hours, the mean and error, and the means and standard deviation; our analysis report is shown in Figure 11.

#### Environmental Measurements

**Figure 12:**
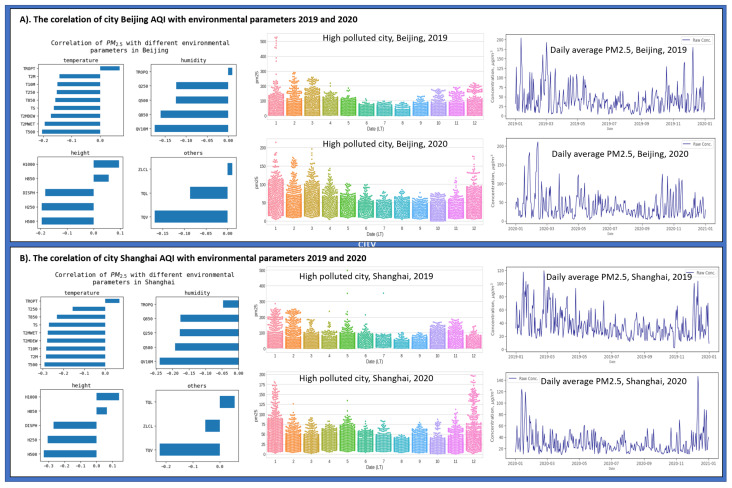
AQI level comparison for eight cities in China and South Korea before and during lockdown.

We discussed the effect of lockdown on the AQI in 2020 after it was implemented in Beijing and Shanghai compared to 2019, where people were spending their time in a routine. It was observed that there is a direct relationship between AQI and people. It was further observed that people travel from one point to another using road vehicles due to routine. Therefore, it can be said that road traffic is in a direct relationship with AQI. If the traffic increases, it increases PM2.5 in the air, and eventually, the AQI level drops to a severe level or even worse in some cities. However, investigating more, we analyzed AQI in correlation with environmental factors, i.e., temperature, humidity, height, and other factors. In Figure 12 it can be seen that from the part that as temperature increases, the PM2.5 decreases, and eventually the AQI level improves. Similarly, humidity and height have an inverse effect on the AQI due to the PM2.5, the primary source affecting the AQI level. It means that if the humidity in the environment is increasing, PM2.5 will decrease. However, high altitude is positively correlated with PM2.5.

## 5. Experiments and Results

This section presents a detailed evaluation of our models. We showed the model efficiency in training accuracy, testing accuracy, recall, precision, F1-score, and confusion matrix. We will also discuss how we have transformed the time-series data to images, and we will also discuss feature extraction. Finally, we present the evaluation of our models and perform comparative analysis with the current state-of-the-art research.

### 5.1. Dataset

**Figure 13:**
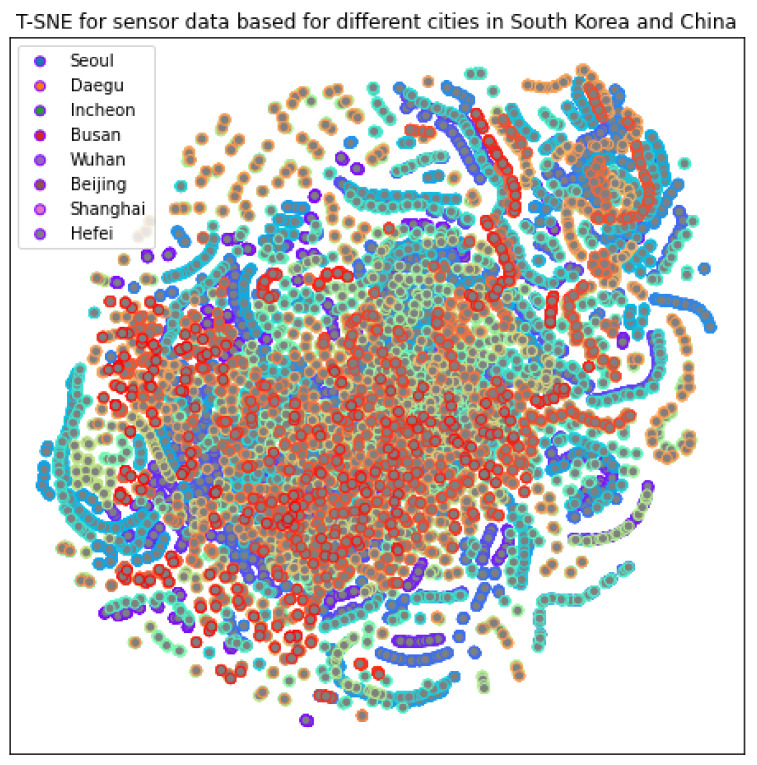
T-SNE for eight cities (Seoul, Daegu, Incheon, Busan, Wuhan, Beijing, Shanghai, Hefei) based on a combination of six sensors (PM2.5, PM10, CO, NO${}_{2}$, SO${}_{2}$, and O${}_{3}$).

We now discuss the dataset that we used for the application of deep learning. For training and testing our model performance, we considered a benchmark dataset that is publicly available from the United States Environment and Protection Agency [41]. This dataset is composed of sensor data from 2015–2020 installed at different locations around the world. We considered the pre-pandemic and pandemic periods of 2019 and 2020 for specific cities, i.e., Seoul, Daegu, Incheon, and Busan from South Korea, and Wuhan, Beijing, Shanghai, and Hefei from China. The sensor data includes data of PM2.5, PM10, CO, NO${}_{2}$, SO${}_{2}$, and O${}_{3}$. The data are sorted in terms of date for the 24 h window. The data also include latitude and longitude information that was fused with the location information. The t-distributed stochastic neighbor-embedding visualization of the dataset is shown in Figure 13.

The sensor data are obtained in time-series format from the real-time IoT system that we have designed as a prototype. Training the deep-learning model, we have considered benchmark data for all sensors. Unfortunately, deep-learning does not work well with time-series data. Therefore, we have produced an innovative technique to initially transform the time-series data into images. After images were obtained, we performed data cleaning. Secondly, we applied a classification methodology to classify the patterns in each city with the combination of patterns from each sensor. Finally, we used the Prophet forecasting model for the forecast of time-series data.

### 5.2. Results and Evaluation

#### 5.2.1. Comparative Evaluation on Model Performance: Cloud vs. Edge

We now describe the evaluation of the proposed deep-learning models (Lw-CNN-1 and Lw-CNN-1) in the cloud (GeForce GTX 1080 Ti) and edge (Jetson Nano) platforms. These two models were built on the cloud platform and evaluated on the edge platform using the input data as follows: (1) the images of the input data were generated as discussed in Section 3.2.2; (2) generation of feature vectors (128 × 128 × 1) from the resized images as discussed in Section 3.2.3; (3) building our 2D-CNN models as discussed in Section 3.3; (4) deployment of these 2D-CNN models to the edge platform as discussed in Section 3.4; (5) inferencing real-time data using these 2D-CNN models as discussed in Section 3.4. This demonstrates collaboration between the cloud and edge platforms in the deep-learning modeling process.

Lw-CNN-1 performs more profound training on the cloud platform and testing on the edge platform compared to Lw-CNN-2. Based on the number of parameters (the size of the models), it can be observed that it takes more time to train (00:09:38 vs. 00:08:41) due to the higher number of trainable parameters (363,882 vs. 132,042). Eventually, it is a more extensive network with a larger size (4.3 MB vs. 1.6 MB) that reports slightly higher accuracy (97.65% vs. 97.19%). On the other hand, Lw-CNN-2 is smaller than Lw-CNN-1 (4.3 MB vs. 1.6 MB). Thus, Lw-CNN-2 is more feasible to be deployed and run on the edge platform with a slight decrease in accuracy (97.65% vs. 97.19%). The inferencing (testing) time of Lw-CNN-2 is also slightly faster (00:00:17 vs. 00:00:09)

**Figure 14:**
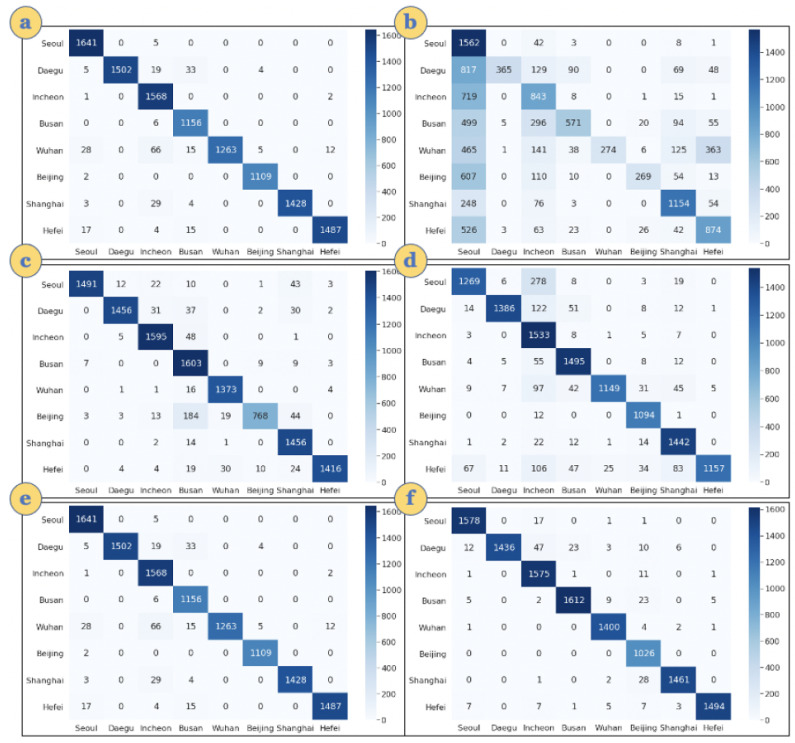
Confusion matrix for AQI event models: (**a**) Lw-CNN-1 on 5 epochs (**b**) Lw-CNN-2 on 5 epochs (**c**) Lw-CNN-1 on 10 epochs (**d**) Lw-CNN-2 on 10 epochs (**e**) Lw-CNN-1 on 15 epochs (**f**) Lw-CNN-2 on 15 epochs.

The size of the models (hyperparameters) and classification training and testing times on the cloud and edge platforms are given in Table 3. The classification accuracy for these two models (Lw-CNN-1 and Lw-CNN-2) is reported for the cloud and edge platforms. As these models are the same for the cloud and edge platforms, there is no difference in accuracy as seen in Table 5. The confusion matrix for these two models based on the number of iterations (epochs) for training is shown in Figure 14.

#### 5.2.2. Deep-Learning Modeling for Edge Intelligence: Lw-CNN-1 vs. Lw-CNN-2

We evaluated the two proposed models (Lw-CNN-1 and Lw-CNN-2) using the following three measures: *Precision* indicates the percentage of the real events out of the total events reported by the model using Equation (13).

(13) $$Precision=\frac{TruePositives}{TruePositives+FalsePositives}$$

*Recall* indicates the percentage of the real events out of the ground-truth events using Equation (14).

(14) $$Recall=\frac{TruePositives}{TruePositives+FalseNegatives}$$

*F1-Score* indicates the harmonic mean of precision and recall using Equation (15).

(15) $$F1-Score=2*\frac{Precision*Recall}{Precision+Recall}$$

We have not observed any issues deploying the models to the edge platform (the Lw-CNN-1 model has only 0.36M parameters and Lw-CNN-2 has 0.13M parameters). However, Lw-CNN-1 performs better with more training because this model is designed for a better learning performance with more trainable parameters than Lw-CNN-2. As seen in Figure 13, the image transformation is very well performed, and all relevant images seem to belong to the same cluster. The Lw-CNN-1 model achieves the highest accuracy of 98.09% for training and 97.65% for testing (from the maximum training with 15 epochs). The lowest accuracy reported by Lw-CNN-1 is 36.25% and 36.81% as its training and testing accuracy (from the minimum training with five epochs), respectively. However, Lw-CNN-2’s most insufficient accuracy is 50.28% for training and 49.97% for testing (from the minimum training with five epochs). In contrast, the highest accuracy is 98.58% for training and 97.19 for testing (from the minimum training with five epochs).

Table 5 shows the training and testing accuracy for Lw-CNN-1 and Lw-CNN-2. We trained our models for 5, 10, and 15 epochs. The training was performed for different epochs to test the model efficiency in terms of the model’s size with a small number of parameters. To validate the results, we have also reported class-wise accuracy based on recall, precision, and F1-score, which can be seen in Table 6.

The classification matrix can be seen in Figure 14a,b for 5 epochs, Figure 14c,d for 10 epochs and Figure 14e,f for 15 epochs, respectively. Each class is divided equally for the classification matrix, but all invalid or unavailable data were filtered out for clean and efficient classification when the models are trained. The classification matrix is also used to compute recall, precision, and F1-score.

#### 5.2.3. Comparative Evaluation with State-of-the-Art Air-Quality Prediction Research

The comparative evaluation with state-of-the-art research in air-quality prediction was also conducted (see Table 7). In our previous work [1], the air-quality level was predicted using three machine-learning algorithms, support vector machine (SVM), random forest, and decision tree with the Seoul AQI dataset (2014–2020) [44].

This current study used the EPA’s AQI dataset [41] including PM2.5, PM10, CO, NO${}_{2}$, SO${}_{2}$, and O${}_{3}$ for 24 h time windows from 2015–2020 in eight cities, i.e., Seoul, Daegu, Incheon, and Busan from South Korea, and Wuhan, Beijing, Shanghai, and Hefei from China. The proposed lightweight CNN models (Lw-1 CNN and Lw-2 CNN) were used for COVID-19 pandemic air-quality event detection for the specific cities from China and South Korea. We have obtained very high prediction accuracy using the proposed deep-learning models. The WRF-Chem model was proposed for the detection of air pollution in urban areas by Xi et al. [20]. They applied traditional machine-learning algorithms, including support vector machines, decision trees, random forests, and gradient boosting, whose precisions ranged from 70 to 85% for the ten leading cities, and from 40 to 53% for the bottom ten cities. A systematic air-quality assessment was undertaken with low-cost sensors across Denmark’s smart cities by Raj et al. [21]. They claimed that SVM outperformed ANN, detecting anomalies at a rate of around 95%. From the power domain, deep-learning models were proposed for power system situation awareness and event investigation, and they improved the detection accuracy by over 48% by Wang et al. [22].

#### 5.2.4. Comparative Evaluation on Cloud and Edge Performance

Evaluation of the proposed event-driven deep-learning models has been performed in both the cloud and edge platforms: (1) NVIDIA GeForce GTX 1080 Ti, (2) NVIDIA Jetson Nano, and (3) Raspberry Pi 4. We used a GPU with 11 GB GDDR5X memory as the cloud platform with the GeForce GTX 1080 Ti. The GPU operates at a frequency of 1481 MHz, running at 1376 MHz (11 Gbps effective). We used the NVIDIA Jetson Nano, a well-known edge device, for prototyping IoT devices for the edge platforms. It has a Quad-core ARM Cortex-A57 MPCore processor CPU, NVIDIA Maxwell w/ 128 NVIDIA CUDA cores GPU and 4 GB 64-bit LPDDR4 Memory. In addition, we used Raspberry Pi 4, with 4GB RAM, a faster quad-core CPU, support for dual displays at up to 4K resolution, Gigabit Ethernet, wireless LAN, Bluetooth 5.0, and USB-C power.

We performed the evaluation and made measurements in terms of time to check the model efficiency. Measurement was performed in seconds. The performance evaluation of all three platforms is shown in Table 8. This table provides information about the data-gathering process, which is recording data from sensors every minute and taking the highest value every hour; eventually, we take 24 readings every day. After data are gathered, we perform data transformation using GAF [38], which is the strategy to transform time-series data into images. When the data are transformed into images, we build our CNN models using the training data (the transformed images) and validate them using the testing data (also transformed image). It is important to note that the performance evaluation table shows the time efficiency for training performed on 15 epochs only. Jetson Nano’s performance is faster than that of Raspberry Pi. Our observations for Jetson Nano and Raspberry Pi are similar to observations reported in a recent study that describes a comparative evaluation of the latency of Jetson Nano and Raspberry Pi 4; Jetson Nano achieved 2.2 s lower latency than Raspberry Pi 4 [45].

## 6. Discussion

Edge intelligence is still in its infancy. As only a few studies are available for AI, there is a limitation to conducting a comparative evaluation for the proposed approach to enable automated prediction and forecasting on real-time events. The system allowed users to be aware of some circumstances of their environment and be notified about the changes. In the Related Work section, we have shown that our work is different from other studies in the areas of edge intelligence, IoT-based real-time event detection, and air-quality prediction using machine learning. In addition, we raised some critical questions regarding the success of edge intelligence. In addition, we tried to answer some of these questions through the proposed event methods, predictive and forecasting models, and air-quality case study for pandemic effects.

- First, what is the most effective way to discover patterns from such large-scale real-time data? We proposed an event-based approach to defining an event of interest by identifying the dominant parameters via correlation analysis to answer this question. The evaluation of the proposed framework has been performed on real-world data of the major cities in China and Korea using event-based deep-learning models to show the robustness and scalability of the proposed model.
- Second, how are they best represented for effective machine learning? We develop a unique way to represent the sensor data in images and overcome the challenges of the real-time processing of data streams in the multi-dimensional domains of time, frequency, and space. The image-based fusion method of sensor or acoustic data has been shown to have superior performance in deep event-driven modeling in our previous studies [46,47,48].
- Third, how can we teach machines to build predictive models for meaningful detection from the massive real-time data? We proposed two unique models for lightweight deep learning for the prediction of air quality in multiple cities on the edge. The proposed deep-learning model is more accurate than traditional machine-learning models, as shown in our previous study [1]. Furthermore, the lightweight design and event-based sensor fusion approach are suitable for edge intelligence.
- Finally, what kind of event-based deep-learning model can be built for edge intelligence? How can we deploy it to the edge? How often do we need to deploy it for real-time event detection? We presented our solutions for the proposed event-based deep-learning models considering real-world events such as the COVID-19 pandemic. For the edge-intelligence research, our best role is to facilitate such innovation for real-time edge intelligence by conducting deep-learning modeling using real-world event scenarios with spatiotemporal context information. However, these solutions have not been thoroughly evaluated.

## 7. Conclusions

This paper proposed a novel framework for event-driven deep-learning edge intelligence, called EDL-EI, which aims to support real-time event detection via the cloud and edge collaboration. The unique contribution of this work is to demonstrate the event-driven deep-learning model via large-sensor fusion using real-world scenarios—air quality and traffic events detected from diverse data from China and South Korea during the COVID-19 pandemic period. These are informative since the significance of the pandemic’s effects and the correlation among parameters are determined based on the long-term and medium-term prediction results of event-based deep-learning models. Some of the research questions on edge intelligence raised in the Discussion section are answered in the current study. The proposed framework showed outstanding predictive accuracy (97.65% and 97.19%) from two deep-learning models on the cities’ air-quality events. Some significant results of the air quality of the major cities of China and South Korea were shown as the effects of the COVID-19 pandemic lockdown.

## Figures and Tables

**Table 1:** Qualitative analysis with related work.

Publication	Model	Methodology	Data	Approach	Limitation
Yang et al. [18]	SVM	Real-time Prediction	Air quality	Real-time air-quality prediction. Predict hourly PM2.5 concentration using Support Vector Regression model	Lack of deep-learning technique. Air-quality detection using a single sensor
Janjua et al. [19]	Feature: LPC, MFCC, and GFCC using Unsupervised Machine learning	IoT Edge-based system	Rare events (gunshot, glass break, siren, and scream)	IoT Edge-based system for rare event detection (gunshot, glass break, siren, and scream)	Detecting rare events without considering the type of event. Overlapping of events detection
Xi et al. [20]	SVM, Decision Trees, Random Forests, Gradient Boosting: Precision	IoT sensor-based detection	74 Chinese cities	Urban Air-Pollution Detection for the Cities in China	Lack of deep-learning techniques. Low performance i.e., low accuracy
Raj et al. [21]	ANN, SVM	IoT sensor-based detection	Denmark’s smart cities’ real-time sensor data	Detecting anomalies using low-cost sensors	Basic neural network model. Not suitable for large datasets
Wang et al. [22]	CNN	Deep-learning-based approach	ROCOF time-series data	Power System Event Detection with CNN fusion	Deep-learning approach not tested with real time

**Table 2:** Hardware components used for air quality.

Sensors/IoT	Devices Used for AQI Detection	Configuration
MQ-5	Natural gas, LPG	Sensing Resistance: 10 KΩ–60 KΩ;
MQ-131	Ground-level ozone	Concentration Scope: 200–10,000 ppm;
MQ-135	CO, ammonia, benzene, alcohol, smoke	Operating voltage: 5 V;
MQ-136	Hydrogen sulfide gas (SO2)	Heater resistance: 33 Ω±5%
PMS5003	Detection of particulate matters (PM10 and PM2.5)	
DHT22	Digital temperature/humidity sensor to measure the surrounding air	Humidity: 0–100% RH; Temperature: −40–−80 °C
Mic	USB microphone	Samsung Galaxy S6
Arduino Uno	Open-source electronic prototyping platform enabling users to create interactive electronic objects	Microcontroller ATmega328P: 8-bit AVR family microcontroller; Input Voltage Limits: 6–20 V; Analog Input Pins: 6 (A0–A5); Digital I/O Pins: 14; DC Current on I/O Pins: 40 mA
Jetson Nano	NVIDIA’s embedded system-on-module and developer kit. Used for real-time machine learning (ML) by deploying ML models to be run on the edge device.	microSD card (16 GB UHS-1 minimum), USB keyboard and mouse, Computer display (either HDMI or DP), Micro-USB power supply (5 V ≡ 2 A)

**Table 3:** Configuration of EDL-EI deep-learning models.

Model	Epochs	#Parameters	Cloud	Edge	Size
			Training Time	Testing Time	
Lw-CNN-1	5	363,882	00:03:48	00:00:14	4.3 MB
	10		00:06:38	00:00:16	
	15		00:09:38	00:00:17	
Lw-CNN-2	5	132,042	00:03:24	00:00:06	1.6 MB
	10		00:05:37	00:00:07	
	15		00:08:41	00:00:09	

**Table 4:** COVID-19 event on air quality and its association in China and Korea.

Event	Effects (Conditions)	Location	Model
Pre-Pandemic	High AQI Level	Seoul, Daegu, Incheon, Busan in South Korea	(1) AQI Level Detection on
Event (2019)	High AQI Level	Wuhan, Beijing, Shanghai, Hefei in China	PM2.5, PM10, CO, O3, NO2, and SO2
Pandemic Event	Moderate AQI Level	Seoul, Daegu, Incheon, Busan in South Korea	(2) Event Detection with Spatial
(2020)	Moderate AQI Level	Wuhan, Beijing, Shanghai, Hefei in China	and Temporal AQI Data
Pre-Pandemic	High road-traffic congestion	Seoul, Daegu, Incheon, Busan in South Korea	(1) Traffic analysis with latitude,
Event (2019)	& poor air quality.	Wuhan, Beijing, Shanghai, Hefei in China	longitude, humidity, temperature, height...
Pandemic Event	Traffic decreased	Seoul, Daegu, Incheon, Busan in South Korea	(2) Correlation of PM2.5 ratio and traffic
(2020)	& air quality improved.	Wuhan, Beijing, Shanghai, Hefei in China	congestion during peak and non-peak hours.

**Table 5:** Model training and testing accuracy based on varying epochs.

Model	Epochs: 5		Epochs: 10		Epochs: 15	
Accuracy	Training	Testing	Training	Testing	Training	Testing
Lw-CNN-1	36.25%	36.81%	94.59%	94.32%	98.09%	97.65%
Lw-CNN-2	50.28%	49.97%	90.08%	88.97%	98.58%	97.19%

**Table 6:** AQI model classification metrics based on recall, precision and F1-score.

Model	Epochs	Metrics	Seoul	Daegu	Incheon	Busan	Wuhan	Beijing	Shanghai	Hefei
Lw-CNN-1	5	Precision	50%	39%	99%	31%	11%	6%	38%	5%
		Recall	93%	82%	19%	58%	97%	100%	96%	100%
		F1-Score	65%	53%	32%	40%	19%	11%	55%	9%
	10	Precision	94%	93%	97%	98%	98%	74%	99%	94%
		Recall	99%	98%	96%	83%	96%	97%	91%	99%
		F1-Score	97%	96%	96%	90%	97%	84%	95%	96%
	15	Precision	100%	96%	100%	100%	91%	100%	98%	98%
		Recall	97%	100%	92%	96%	100%	99%	100%	99%
		F1-Score	98%	98%	96%	98%	95%	100%	99%	98%
Lw-CNN-2	5	Precision	97%	24%	53%	37%	19%	25%	75%	56%
		Recall	29%	98%	50%	77%	100%	84%	74%	62%
		F1-Score	44%	39%	51%	50%	32%	39%	75%	59%
	10	Precision	80%	87%	98%	95%	83%	99%	97%	76%
		Recall	93%	98%	69%	90%	98%	91%	89%	99%
		F1-Score	86%	92%	81%	92%	90%	95%	93%	86%
	15	Precision	99%	93%	99%	97%	99%	100%	98%	98%
		Recall	98%	100%	96%	98%	99%	92%	99%	100%
		F1-Score	99%	97%	97%	98%	99%	96%	99%	99%

**Table 7:** State-of-the-art research on machine learning for air-quality detection.

Event Prediction (Ours)	EPA’s AQI Dataset [41]	Lw-CNN-1: 97.65%, Lw-CNN-2: 97.19%	Air-Quality Event Detection (City and Time)
Shah et al. (2020) [1]	Seoul AQI dataset (2014–2020) [44]	Decision Tree 76%, Random Forest 82%, SVM 95%	Air-quality level Prediction
Xi et al. (2015) [20]	74 Chinese cities	SVM, Decision Trees, Random Forests, Gradient Boosting: Precision 70–85% (10 cities)	Urban Air-Pollution Detection for the Cities in China
Raj et al. [21]	Denmark’s smart cities’ real-time sensor data	ANN, SVM: Accuracy 95%	Detecting anomalies using low-cost sensors
Wang et al. (2020) [22]	ROCOF time-series data	CNNs accuracy 94.42%	Power System Event Detection

**Table 8:** Edge intelligence: event-based deep-learning (DL) performance (time in seconds).

	Device	Data-Gathering	Data Transformation Time (s)	DL Model	Training Time (s)	Testing Time (s)
Cloud	NVIDIA	Record every	342	Lw-CNN-1	468	12
	1080 Ti GPU	minute from		Lw-CNN-2	414	7
Edge	NVIDIA Jetson	sensors and select	702	Lw-CNN-1	558	17
	Nano (proposed)	the highest value		Lw-CNN-2	504	9
	Raspberry Pi 4	from 24 readings	936	Lw-CNN-1	756	22
		every day		Lw-CNN-2	642	19

Please note that for the cloud performance, we have not included the time for communication between the cloud and the edge platforms.

## Data Availability

The link to publicly archived datasets analyzed is https://github.com/khushalshah1989/EDL-EI/blob/main/README.md. The link to the data we also generated during the study is https://github.com/khushalshah1989/EDL-EI/upload/main/Data/img.
